# Impact of hydrophilic side chains on the thin film transistor performance of a benzothieno–benzothiophene derivative†

**DOI:** 10.1039/d4ma00594e

**Published:** 2024-07-10

**Authors:** Mindaugas Gicevičius, Ann Maria James, Lukas Reicht, Nemo McIntosh, Alessandro Greco, Lamiaa Fijahi, Félix Devaux, Marta Mas-Torrent, Jérôme Cornil, Yves Henri Geerts, Egbert Zojer, Roland Resel, Henning Sirringhaus

**Affiliations:** a Optoelectronics Group, Cavendish Laboratory, University of Cambridge JJ Thomson Avenue Cambridge CB3 0HE UK mg980@cam.ac.uk hs220@cam.ac.uk; b Institute of Solid State Physics, NAWI Graz, Graz University of Technology Petersgasse 16 8010 Graz Austria; c Laboratory for Chemistry of Novel Materials, University of Mons 7000 Mons Belgium; d Max Planck Institute for Polymer Research 55128 Mainz Germany; e Institut de Ciència de Materials de Barcelona, ICMAB-CSIC, Campus de la UAB 08193 Bellaterra Spain; f Laboratoire de Chimie des Polymères, Faculté des Sciences, Université Libre de Bruxelles (ULB) CP 206/1, Boulevard du Triomphe 1050 Bruxelles Belgium; g International Solvay Institutes of Physics and Chemistry, Université Libre de Bruxelles 1050 Bruxelles Belgium

## Abstract

Side-chain engineering in molecular semiconductors provides a versatile toolbox for precisely manipulating the material's processability, crystallographic properties, as well as electronic and optoelectronic characteristics. This study explores the impact of integrating hydrophilic side chains, specifically oligoethylene glycol (OEG) units, into the molecular structure of the small molecule semiconductor, 2,7-bis(2(2-methoxy ethoxy)ethoxy) benzo[*b*]benzo[4,5] thieno[2,3-*d*] thiophene (OEG-BTBT). The investigation includes a comprehensive analysis of thin film morphology and crystallographic properties, along with the optimization of deposition parameters for improving the device performance. Despite the anticipated benefits, such as enhanced processability, our investigation into OEG-BTBT-based organic field-effect transistors (OFETs) reveals suboptimal performance marked by a low effective charge carrier mobility, a low on/off ratio, and a high threshold voltage. The study unveils bias stress effects and device degradation attributed to the high ionization energy of OEG-BTBT alongside the hydrophilic nature of the ethylene–glycol moieties, which lead to charge trapping at the dielectric interface. Our findings underscore the need for a meticulous balance between electronic properties and chemical functionalities in molecular semiconductors to achieve stable and efficient performance in organic electronic devices.

## Abstract 2

Side-chain engineering in molecular semiconductors provides a versatile toolbox for precisely manipulating the material's processability, crystallographic properties, as well as electronic and optoelectronic characteristics.

## Graphical abstract

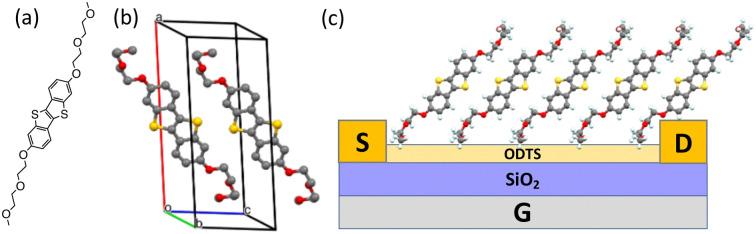

## Introduction

Organic electronics is a rapidly evolving field with unparalleled potential for a wide range of applications, including organic photovoltaics (OPVs), organic light-emitting diodes (OLEDs), and organic field-effect transistors (OFETs).^1–3^ The diverse and versatile nature of organic compounds enables precise tailoring of their properties, offering a robust platform for charge transport studies and device engineering. This tunability extends from the molecular level, where functional groups and conjugation lengths can be judiciously selected, to the macroscopic scale, where film morphology and device architecture can be tuned.^4,5^

Among the rich variety of organic semiconductors (OSCs), small molecule semiconductors with thienoacene-based cores have been established as a class of best-performing materials^6^ with charge carrier mobilities routinely exceeding 10 cm^2^ V^−1^ s^−1^.^7,8^ This outstanding property has made them the model systems for studying both charge and thermal transport mechanisms as well as for understanding structure–property relationships in crystalline OSCs.^9–11^

One promising avenue in the quest for improving the functionalities of high-performance molecular semiconductors involves the incorporation of hydrophilic side chains into their molecular structures. The integration of hydrophilic moieties not only enhances the solubility and processability of these materials in green solvents but also opens intriguing opportunities to tailor their electronic and optoelectronic properties.^12^ Previous studies have shown that the incorporation of oligoethylene glycol (OEG) units is of particular interest for mixed ionic–electronic conductors,^13^ while in small molecule semiconductors it has been shown to boost thermoelectric properties by increasing the electric conductivity while keeping the material's thermal conductivity low.^14^ Furthermore, it has been shown that oligoethylene glycol substituents improve the performance of not only n-type OSCs but also has the potential of increasing the hole mobility in p-type molecular semiconductors.^15^

In this study, we focus on a novel molecular semiconductor 2,7-bis(2(2-methoxy ethoxy)ethoxy) benzo[*b*]benzo[4,5] thieno[2,3-*d*] thiophene (OEG-BTBT), which is based on a high-mobility BTBT core with 2,7-(oligoethylene glycol) substituents. The OEG side chains possess enhanced hydrophilicity, ionic conductivity, high polarity, and flexibility. Thus, their study offers interesting insights into the impact of the functionalization of conjugated BTBT cores. In particular, it is interesting to understand how hydrophilic OEG sidechains influence the charge transport properties in OFET devices and how these differ from the properties of widely studied high-performance, alkyl-substituted BTBT derivatives. We investigated bottom-gate OFETs based on OEG-BTBT thin films deposited by physical vapor deposition and carried out an extensive analysis of the deposited films using crystallographic, spectroscopic, as well as scanning probe techniques, combined with quantum-mechanical simulations. These studies were combined with a careful optimization of OFET devices to identify the key factors affecting the device performance.

## Experimental details

Synthesis and purification of 2,7-bis(2(2-methoxy ethoxy)ethoxy)benzo[*b*]benzo[4,5] thieno[2,3-*d*]thiophene (OEG-BTBT) have been reported previously.^16^ Bottom-gate/bottom-contact (BGBC) OFETs with interdigitated source and drain electrodes (*W* = 1000 μm, *L* = 5 and 10 μm) were prepared on *n*^++^ Si/SiO_2_ (300 nm, *C*_i_ = 11.5 nF cm^−2^) substrates by double-layer liftoff UV photolithography. Source/drain contacts (Cr/Au or Ti/Au, 3/25 nm) were deposited by thermal evaporation, whereas CrNi/Pt electrodes were deposited by RF sputtering. The modification of Au electrodes with pentafluorobenzenethiol (PFBT) self-assembled monolayers was carried out by immersing the samples in 1 mM PFBT solution in ethanol for 1 hour at 75 °C, followed by rinsing with ethanol and drying with N_2_. Octadecyltrichlorosilane (ODTS) treatment of the SiO_2_ surface was subsequently carried out by treating samples with 1 mM ODTS solution at 60 °C for 30 minutes in toluene. The homogeneity of the ODTS layer was checked by X-ray reflectivity, with a measured layer thickness of 1.6 nm, corresponding to the thickness of a single ODTS monolayer.

Thin layers of OEG-BTBT with a nominal thickness of 30 nm were deposited by thermal evaporation (CreaPhys GmbH) at a deposition rate of 1.2 nm min^−1^ under a pressure of 2 × 10^−6^ mbar. During the deposition the substrates were rotated at 10 rpm and were maintained at a specific set temperature. All sample handling of OEG-BTBT OFET devices was conducted under an inert N_2_ atmosphere with H_2_O and O_2_ concentrations <5 ppm to minimize atmospheric exposure of the OEG-BTBT films.

Effective charge carrier mobility values in OFET devices were extracted from the forward scan of the transfer curves in the saturation regime using the following equation:1
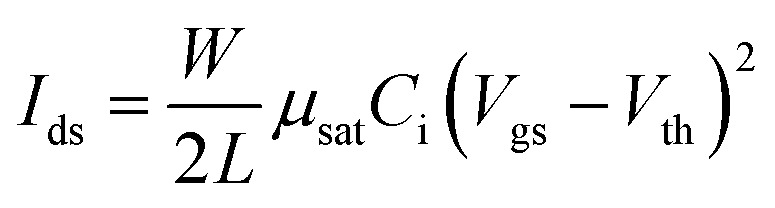
where *I*_ds_ is the drain current, *W* and *L* are transistor channel width and length, *V*_gs_ and *V*_th_ are the gate and threshold voltages, and *C*_i_ is the areal capacitance of the dielectric.

Atomic force microscopy (AFM) measurements were performed under ambient conditions in tapping mode using an Asylum Research MFP-3D AFM System (Oxford Instruments) equipped with TESPA-V2 probes (Bruker) with a nominal tip radius of 7 nm, a resonant frequency of 320 KHz, and a spring constant of 37 N m^−1^.

Low-frequency Raman spectroscopy measurements were performed in back-scattering geometry using a HORIBA Jobin Yvon T64000 triple grating spectrometer with a 633 nm HeNe laser excitation source. Raman spectroscopy measurements were performed under ambient conditions. The power of the excitation laser power was kept below 5 mW to avoid sample degradation and was focused to a spot approximately 2 μm in diameter through a 100×/0.9 NA objective.

The transfer Integrals were computed at the density functional theory (DFT) level with the ADF package^17^ by a fragment orbital approach,^18^ using the B3LYP functional^19^ and the DZ basis set.^20^

DFT simulations of the Raman spectrum of crystalline OEG-BTBT were performed with the Vienna *Ab initio* Simulation Package (VASP) Version 5.4.4^21^ using the PBE functional^21^ and the Grimme D3 van der Waals correction with Becke–Johnson damping.^22–24^ The simulations were based on the crystal structure of OEG-BTBT taken from the Cambridge Structural Database (deposition number 2109678).^25^ The positions of the atoms were relaxed until all forces fell to below 0.001 eV Å^−1^. In passing we note that a full optimization including a relaxation of the lattice parameters yielded a Raman spectrum in worse quantitative agreement with experiment than when adopting the experimental unit-cell parameters, as discussed in the ESI.† This is attributed to the neglect of thermal expansion in the DFT optimized unit cell. The plane-wave energy cut-off defining the used basis set was set to 900 eV and a 1 × 2 × 2 *k*-point grid was used to sample reciprocal space. These settings converge the total energy per atom to 0.5 meV, as shown in the ESI.†

The properties of the *Γ*-point phonons were calculated using a finite-displacements scheme as implemented in phonopy,^26^ displacing each atom by 0.01 Å in every Cartesian direction. In previous studies, we found this approach to provide an excellent agreement between calculated and experimental Raman spectra concerning peak positions also in the low-frequency lattice phonon region with average deviations typically below 7 cm^−1^.^27,28^ To confirm the negligible role played by anharmonicities, calculations with displacements of 0.005 Å and 0.02 Å were also performed yielding equivalent results (see ESI,† where also a more in-depth analysis of potential anharmonicities is provided).

The same displaced structures were used for calculating the Raman tensor employing the approach described in ref. 29, which significantly reduces the computational cost compared to conventional schemes calculating the Raman tensor by displacing the structure along eigenmodes. To convert this into a tensor of Stokes intensities, the following equation was used^30^2
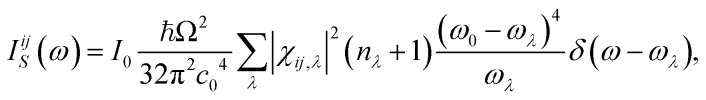


With the intensity of the incident radiation *I*_0_, the sample scattering volume Ω, the speed of light in vacuum *c*_0_, the Raman tensor *χ*_*ij*,*λ*_, the Bose–Einstein distribution *n*_*λ*_, the frequency of the incident light *ω*_0_, and the phonon frequency of phonon mode *λ*, *ω*_*λ*_. As we are only interested in relative peak intensities, the choice of the values of *I*_0_ and Ω is inconsequential. Indices *i* and *j* denote cartesian directions. The temperature was set to 300 K and the wavelength of the excitation laser to 633 nm. As the experimental sample was polycrystalline with no apparent texture within the plane of the thin film, we refrained from considering the exact scattering geometry and obtained the Raman spectrum by averaging over all orientations *via* an integral over the three Euler angles, as described in ref. 31. The spectrum was then constructed as a sum of Lorentzian functions centred at the frequencies of the Raman-active modes with amplitudes corresponding to the Stokes and broadened by 0.1 THz (full width at half maximum of the Lorentzian).

Combined X-ray reflectivity (XRR) and X-ray diffraction (XRD) measurements were performed on a PANalytical Empyrean diffractometer in *θ*–*θ* geometry using CuKα radiation operating at a wavelength of *λ* = 1.542 Å. The primary side was equipped with a sealed copper tube and a multilayer mirror to produce a parallel beam. The secondary side had an anti-scatter slit, a 0.02 rad Soller slit, and the PANalytical PIXcel 3D detector operating as a point detector. The angular measurements (2*θ*) were converted to reciprocal space using *q*_*z*_ = 4π/*λ* sin *θ*, where *q*_*z*_ is the scattering vector perpendicular to the substrate. The observed Bragg peaks were analysed in terms of peak positions, where the corresponding interplanar distance (*d*) of a net plane series can be calculated on the basis of *q*_*z*_ by *d* = 2π/*q*_*z*_. The vertical crystal size *L*_*z*_ is related to the peak width Δ*q*_*z*_*via L*_*z*_ = 2π/Δ*q*_*z*_.

## Results

**Fig. 1:**
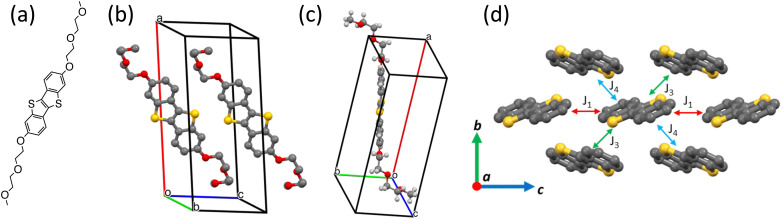
(a) Molecular structure of OEG-BTBT. (b) Molecular packing and (c) conformation of OEG-BTBT in the crystallographic unit cell. (d) Notation of the transfer integrals between the highest occupied molecular orbitals (HOMO) of neighbouring BTBT units within the herringbone arrangement. The side chains of OEG-BTBT were removed for clarity.

OEG-BTBT is a solution-processable OSC composed of a high-mobility benzothieno–benzothiophene (BTBT) core with oligo(ethylene glycol) (OEG) substituents at its terminal ends (Fig. 1(a)). The initial step in evaluating charge transport properties in OEG-BTBT was to consider its molecular packing in the solid-state, which significantly influences the associated electronic properties. The crystal structure of OEG-BTBT was published recently and the arrangement of the molecules within the crystallographic unit cell is shown in Fig. 1(b).^25^

The molecular packing of OEG-BTBT is comparable to other substituted BTBT-type molecules, with BTBT cores arranged in planes separated by the layers of OEG side chains, thus creating a lamellar structure. The OEG chains adopt an angled conformation dictated by the interaction between oxygen and hydrogen atoms of the neighbouring molecules (Fig. 1(c)). The conjugated BTBT units are arranged in a herringbone pattern along the crystallographic (100) plane. Due to the herringbone type stacking of the BTBT units, it can be anticipated that the material exhibits a 2-dimensional charge transport behaviour.^32^

Based on the crystal structure, the transfer integrals between the highest occupied molecular orbitals (HOMO) of next neighbours were calculated within the herringbone plane, as depicted in Fig. 1(d). We calculated the following values of transfer integrals: *J*_1_ = 21.4 meV, *J*_3_ = 12.2 meV, and *J*_4_ = 20.7 meV, adhering to the nomenclature commonly used for BTBT-type molecules.^33,34^ The reorganization energy for BTBT compounds is on the order of ∼200–250 meV^17^ which meets the condition *λ*/4 > *J* that entails a more hopping-like transport.^35^ Thus, the sign of the transfer integrals becomes irrelevant since the hopping rate relies on the Marcus equation featuring the square of the electronic coupling:3
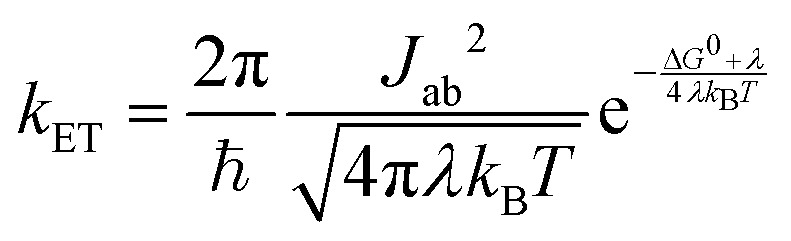


The calculated transfer integral values for OEG-BTBT are approximately 3 times lower compared to the alkyl-substituted BTBT-derivatives.^9^ These differences likely arise from the distinct packing of BTBT cores dictated by the different nature of side chains.

In the subsequent step, the energy levels of frontier molecular orbitals in OEG-BTBT were determined. The incorporation of electron donating substituents with the oxygen atoms directly connected to the BTBT core has been previously shown to decrease the first oxidation potential of from 0.824 V in 2,7-dioctyl BTBT to 0.507 V for 2,7-dioctyloxy-BTBT, *vs.* Fc/Fc^+^.^36^ However, in OEG-BTBT thin films, the reported ionization energy (IE) value is 5.54 ± 0.03 eV, which is similar to other BTBT molecules with alkyl and alkoxy substituents.^16,33,36^ This similarity suggests that the electron-donating effects, which typically lower the IE, are diminished due to the less favorable crystal packing in alkoxy-substituted BTBT derivatives. We measured the optical band gap value of 3.63 eV in OEG-BTBT using UV-Vis spectroscopy in solution (ESI,† Fig. S1), allowing us to make a rough estimate for the electron affinity of −1.9 eV. The similar values of the transfer integrals within the BTBT planes together with the positions of the frontier states relative to the vacuum level make OEG-BTBT a reasonable candidate for transistor applications.^37^

An essential step in realizing OFET devices based on OEG-BTBT involves optimizing the thin film morphology and crystallographic properties. Due to the low solubility of OEG-BTBT in common solvents (the highest solubilities were found to be 0.9 g L^−1^ in dichloromethane and 0.7 g L^−1^ in chloroform)^38^ it was not feasible to prepare films with sufficient thickness and homogeneity for transistor applications using solution-based methods. In fact, we have made efforts to deposit OEG-BTBT films from solution using spin-coating and bar-assisted meniscus shearing techniques, but the resulting films did not exhibit field-effect modulation of channel conductivity. For these reasons, the deposition of OEG-BTBT thin films was carried out by physical vapor deposition.

**Fig. 2:**
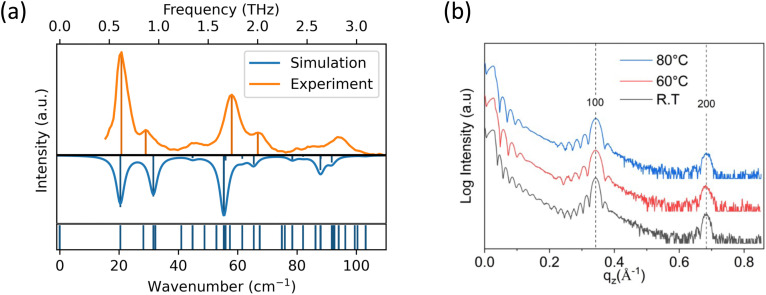
(a) Measured low-frequency Raman spectrum of an OEG-BTBT film in combination with a theoretical calculation of the Raman spectrum based on the thermodynamically stable phase of OEG-BTBT. The four main peaks in the experiment are indicated by vertical bars. Simulated Raman-active modes are indicated by bars on the negative scale in the upper panel. The lower panel illustrates the frequencies of all modes in the chosen range (including Raman-active and Raman-inactive ones). (b) Combined X-ray reflectivity and X-ray diffraction of 30 nm OEG-BTBT films prepared by physical vapor deposition at different substrate temperatures. Vertical lines give the expected positions of Bragg peaks together with their indices.

In previous studies, OEG-BTBT has been shown to crystallize in different polymorphic states, thus it was necessary to determine the crystallographic structure of vacuum-deposited films.^25,38^ For this, we used low-frequency Raman spectroscopy in a spectral region dominated by intermolecular vibrations, which displays high sensitivity towards intermolecular ordering. This makes low-frequency Raman spectroscopy a valuable technique for differentiating between different crystal packings, especially as specular X-ray diffraction can suffer from the problem that different polymorphs might exhibit hard to distinguish X-ray diffraction patterns.^39,40^ The results of Raman spectroscopy are depicted in Fig. 2(a) along with a calculated Raman spectrum based on the known thermodynamically stable structure of OEG-BTBT with deposition number 2109678 in the Cambridge Structural Database.^25^ The selected low wavenumber range of the Raman spectra facilitated clear phase identification. The excellent agreement between the experimental and the simulated spectrum suggests that the investigated sample indeed crystallizes in the thermodynamically stable bulk phase of OEG-BTBT employed in the simulations. To quantify this agreement, we calculated the root-mean-square deviation of the four main peaks (at 19, 28, 56 and 65 cm^−1^) to be 2.0 cm^−1^, which corresponds to 0.25 meV. As discussed in ref. 28,41, much more significant frequency shifts between theory and experiment are expected when dealing with different polymorphs. The larger deviations between measured and calculated peak intensities do not impact the polymorph identification, as they are primarily a consequence of not accounting for the texture of the film in the simulations.

In the subsequent step, we investigated the structural properties of thin films prepared at different substrate temperatures (23 °C, 60 °C and 80 °C) through combined X-Ray reflectivity (XRR) and X-Ray diffraction (XRD) analyses (Fig. 2(b)). The diffraction patterns show the 100 Bragg peak, positioned at *q*_*z*_ = 0.34 Å^−1^, and its higher order reflection at *q*_*z*_ = 0.68 Å^−1^. An interplanar distance of 18.3874 Å was determined, which is in excellent agreement with the thermodynamically stable bulk phase of OEG-BTBT, further confirming the polymorph identification. Notably, identical interplanar distance values were observed for the OEG-BTBT films deposited on SiO_2_ and ODTS-modified SiO_2_ substrates. The observation of only (ℓ00) peaks indicates a strong preferred orientation of the OEG-BTBT crystallites with the (ℓ00) planes aligned parallel to the substrate surface. This crystal orientation implies that the herringbone layers of the BTBT units are also oriented parallel to the substrate surface, which is a preferred direction for charge transport and for employing such films in OFET devices.

A more detailed examination of the diffraction pattern reveals Kiessig fringes below *q*_*z*_ ∼ 0.2 Å^−1^. Fitting the experimental data yields an average layer thickness of 25 nm, 22 nm and 24 nm for the films prepared at 23 °C, 60 °C and 80 °C, respectively. Additionally, the Laue fringes around the 100 Bragg peak reveal crystals with homogenous extensions of the crystalline domains perpendicular to the substrate surface. Based on the width of the central part of the Bragg peak, vertical (out-of-plane) crystal sizes of 26 nm, 23 nm and 27 nm are found for the films deposited at 23 °C, 60 °C and 80 °C, respectively. The excellent agreement between the film thickness and vertical crystal extension indicates the presence of single crystalline domains throughout the entire thickness of the film.

**Fig. 3:**
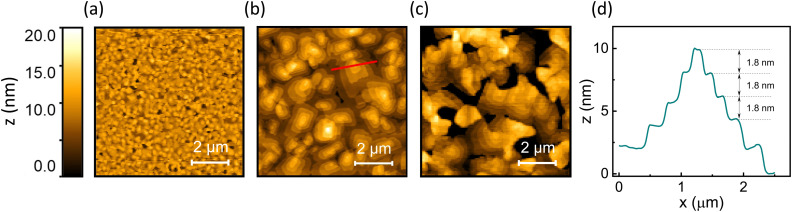
AFM topography images of OEG-BTBT thin films deposited on SiO_2_/ODTS at (a) 23 °C, (b) 60 °C, and (c) 80 °C. (d) AFM height profile of a crystal domain in the OEG-BTBT thin film deposited at 60 °C (marked by a red line in panel (b)), showing a step height of 1.8 nm corresponding to the interplanar distance in the thermodynamically stable bulk phase.

While the average film thickness and the vertical extent of the crystallites have been shown to not depend on the substrate temperature during deposition, the temperature plays a crucial role in controlling the thin film morphology and the horizontal extent of the crystallites. This is revealed by atomic force microscopy (AFM) investigations of OEG-BTBT films deposited at different temperatures (23 °C, 60 °C, and 80 °C). Films deposited with substrate held at room temperature exhibited a small lateral grain size and a root mean square (RMS) roughness of 2.0 nm (Fig. 3(a)). A substantial improvement in thin film morphology was observed when the deposition of OEG-BTBT was carried out at an elevated substrate temperature of 60 °C. Crystal domains with significantly larger lateral dimensions (>1 μm) were formed while maintaining the RMS roughness of 2.2 nm (Fig. 3(b)). Fig. 3(d) shows the AFM height profile of the OEG-BTBT crystal domain for the film deposited at 60 °C. It demonstrates a terraced morphology with a typical step height of 1.8 nm. This step height is in excellent agreement with the interplanar distance 18.3874 Å of the thermodynamically stable bulk phase of OEG-BTBT.

Notably, while the AFM data depict the surface topography of the OEG-BTBT films and reveal the lateral extension of the crystallites, they do not provide information on the thickness of the film or the underlying film morphology underneath the observed terraces. However, a previous study on thin film growth of OEG-BTBT deposited by thermal evaporation has shown that in the initial stage of film growth a closed monolayer is formed, followed by layer-by-layer growth and the formation of crystalline islands, indicative of Stranski–Krastanov growth.^42^

For the last sample, which is shown in Fig. 3(c), a further increase in the substrate temperature to 80 °C leads to the formation of disconnected domains, pinholes, and an increased surface roughness with an RMS value of 7.4 nm. The above data show that the best film continuity in combination with a large lateral grain size of the OEG-BTBT (>1 μm) is obtained for films deposited at 60 °C. Thus, thin films deposited at 60 °C were selected for OFET device preparation, since in such films charge transport is expected to be least affected by carrier scattering and/or trapping at grain boundaries and crystal step edges.^43^

**Fig. 4:**
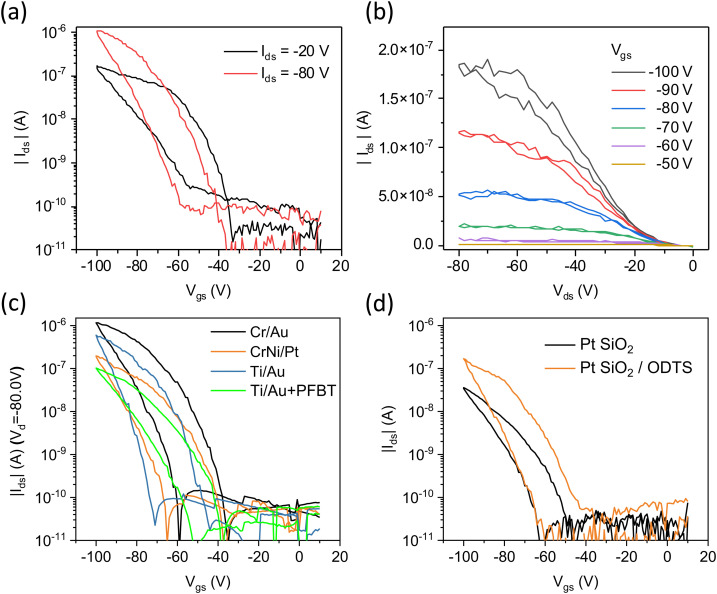
Transfer (a) and output (b) characteristics of OEG-BTBT based thin film transistors with Cr/Au source/drain electrodes in the linear and saturation regimes. (c) Transfer characteristics of OEG-BTBT OFET devices with different source/drain electrodes. (d) Transfer characteristics of OEG-BTBT-based transistors with Pt electrodes on an untreated SiO_2_ dielectric and a SiO_2_ dielectric modified with *n*-octadecyltrichlorosilane (ODTS) self-assembled monolayer. Transistor channel length of 5 μm.

Fig. 4(a) displays typical transfer characteristics of a bottom-gate, bottom-contact (BGBC) OEG-BTBT OFET device with Cr/Au contacts and an ODTS self-assembled monolayer at the surface of the dielectric. The schematics of the device architecture and electrode layout are presented in the ESI† in Fig. S2. OEG-BTBT transistor devices with a bottom-gate, top-contact (BGTC) architecture were also fabricated but did not demonstrate the field-effect modulation of the channel conductivity. The transfer curves show p-type transport characteristics of OEG-BTBT-based BGBC OFET device with an *I*_on_/*I*_off_ ratio of 10^4^. The presence of pronounced non-idealities in the OEG-BTBT transistor performance are evidenced by a substantial threshold voltage *V*_th_ and by a significant hysteresis. Fig. 4(b) shows the output characteristics of OEG-BTBT OFET device with prominent non-linearities at low drain voltages, indicating a high contact resistance.

The performance of OFET devices largely relies on the efficient injection and extraction of charges to/from the active channel, which is governed by the alignment of the Fermi-level of the contact metal (determined by its work function (*ϕ*)) and the transport level for holes in the p-type OSC. To investigate whether the performance of OEG-BTBT transistors is limited by the metal/OSC interface effects, we fabricated OFET devices using modified contacts. In addition to gold, we employed the larger work function metal platinum, and, most importantly, Au modified with a pentafluorobenzenethiol (PFBT) self-assembled monolayer (*ϕ* = 5.6 eV). This is expected to provide a significantly better alignment of the Fermi level of the contact metal with the hole transport level of OEG-BTBT.^44^ The transfer characteristics of OEG-BTBT-based OFET devices with different source/drain electrode materials are depicted in Fig. 4(c). They show that varying the source/drain electrode in OEG-BTBT-based OFET devices results in only a marginal difference in device performance with no significant improvement in threshold voltage and hysteresis.

Further device characteristics are summarized in Table 1, indicating no substantial increase in the *I*_on_/*I*_off_ ratios and effective charge carrier mobilities by changing/modifying the electrode. These findings suggest that the primary limitations to the OEG-BTBT OFET device performance do not arise from poor charge injection/extraction due to an energy level misalignment at the metal/OSC interfaces.

**Table 1:** Comparison of OEG-BTBT transistor performance characteristics of devices prepared with different configurations of source/drain electrodes and dielectric interfaces. Transistor channel length of 5 μm

S/D metal	Surface dielectric	*V*_th_ (V)	*I*_on_/*I*_off_	*μ*_sat_ (cm^2^ V^−1^ s^−1^)
Cr/Au	ODTS	−54	∼1.5 × 10^4^	6.0 × 10^−4^
Ti/Au	ODTS	−59	∼1.9 × 10^4^	3.6 × 10^−4^
Ti/Au + PFBT	ODTS	−54	∼1.8 × 10^3^	6.2 × 10^−5^
CrNi/Pt	ODTS	−52	∼3.1 × 10^3^	8.0 × 10^−5^
CrNi/Pt	SiO_2_	−65	∼1.4 × 10^3^	2.5 × 10^−5^

In OFET devices, charge accumulation primarily occurs within the first few layers of OSC at the interface with the gate dielectric, making charge carrier mobility and threshold voltage highly susceptible to the surface chemistry of the gate dielectric/OSC interface.^45,46^ For instance, it has been shown that water adsorption at the surface of thermally grown silicon oxide may lead to the formation of trap states.^47^ Approaches to mitigate charge trapping often involve modifying the dielectric interface through the introduction of self-assembled monolayers (SAM), such as *n*-octadecyltrichlorosilane (ODTS), which aim to passivate potential trap sites. Additionally, they can make the surface more hydrophobic, thus reducing adsorption of water molecules.^16^ Notably, the previously described OFET devices already contained a surface modification of the SiO_2_ dielectric with *n*-octadecyltrichlorosilane (ODTS) self-assembled monolayers. Fig. 4(d) illustrates the impact of ODTS modification on OEG-BTBT transistor performance, demonstrating a 3 times higher effective charge carrier mobility and *I*_on_/*I*_off_ ratio in OFET devices with CrNi/Pt contacts and ODTS self-assembled monolayers. This effect was even more pronounced in devices featuring Ti/Au contacts (ESI,† Fig. S3). The quality of ODTS self-assembled monolayers was investigated using X-ray reflectivity measurements, revealing a layer thickness of 1.6 nm, and indicating an excellent coverage of the underlying SiO_2_ substrate (ESI,† Fig. S4).

**Fig. 5:**
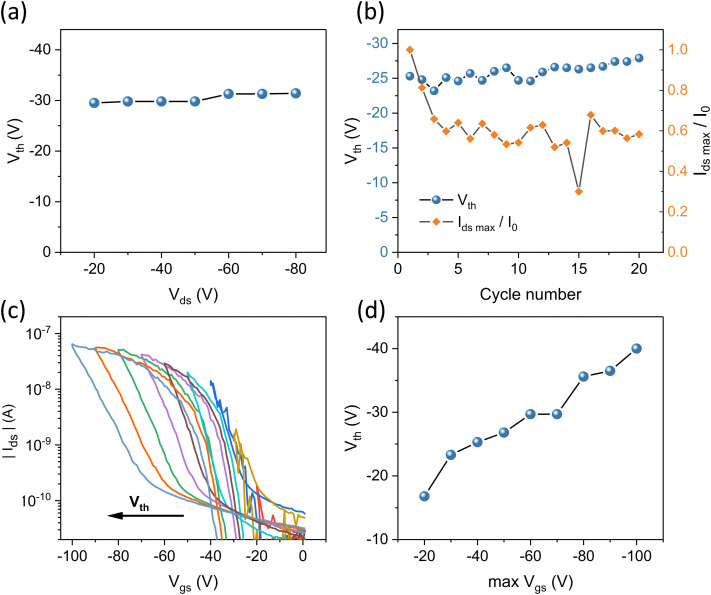
(a) Evolution of threshold voltage *V*_th_ upon increasing maximum source–drain voltage *V*_ds_, measured at *V*_gs_ = −40 V in OFET device with Ti/Au source drain contacts (*L* = 10 μm). (b) The change of maximum source–drain current *I*_ds max_ (*V*_gs_ = −40 V, *V*_ds_ = −10 V) and of the threshold voltage *V*_th_ as a function of repeated transistor switching (*L* = 10 μm). (c) Transfer curves of OEG-BTBT transistor device at increasing maximum gate voltages and *V*_ds_ = −10 V, showing an increase in threshold voltage *V*_th_ upon the increase of maximum *V*_gs_ (*L* = 10 μm). (d) The change in *V*_th_*versus* the maximum *V*_gs_.

The above data show that the passivation of the dielectric surface did not resolve the strongly negative threshold voltages in OEG-BTBT-based OFET. Thus, to identify their origin, we more thoroughly investigated the effects of bias stress in the freshly fabricated OFET devices with Ti/Au contacts and ODTS self-assembled monolayer. Taking into account that relatively high source–drain and source–gate voltages were applied, we examined the effects of source–drain and source–gate voltages separately. Firstly, we examined how the threshold voltage *V*_th_ is affected by an increase in the absolute value of the maximum source drain voltage *V*_ds_ up to −80 V. This was done, while recording the transfer curves in a *V*_gs_ range between *V*_gs_ = +10 V and a moderately low *V*_gs_ = −40 V (scan rate of 4 V s^−1^). The result is depicted in Fig. 5(a), revealing no dependence of *V*_th_ on the maximum applied *V*_ds_. These observations demonstrate that the transistor performance shows no apparent degradation induced by high source–drain voltage.

We then investigated the effects of repeated transistor operation by repeatedly recording dual-sweep transfer curves between *V*_gs_ = +10 V and *V*_gs_ = −40 V at the scan rate of 4 V s^−1^, while applying a low source/drain voltage of *V*_ds_ = −10 V (Fig. 5(b)). After the first three switching cycles the maximum drain current decreases by about 35 percent and then reaches a plateau, demonstrating a stable performance upon repeated switching. In contrast, the *V*_th_ remains largely unaffected by the repeated switching of the OFET device, increasing by only −2.5 V after 20 cycles.

Finally, we investigated how *V*_th_ is influenced by an increase in maximum applied gate bias. We recorded transfer curves with progressively higher absolute value of the maximum gate voltage. These reveal an apparent increase in both *V*_th_ and the hysteresis (Fig. 5(c)). Fig. 5(d) shows a relatively small initial *V*_th_ of −17 V at low *V*_gs_, which increases rapidly when a higher gate bias is applied. Furthermore, the increase in *V*_th_ is also accompanied by a strong increase in hysteresis. This observation indicates that OFET channel degradation primarily occurs at the OEG-BTBT/dielectric interface.

## Discussion

We investigated the charge transport properties of thermally evaporated OEG-BTBT thin films in bottom-gate/bottom-contact (BGBC) field-effect transistor devices. The transistor characteristics exhibited significant non-idealities, including a pronounced hysteresis, high threshold voltages, and low effective carrier mobilities. Common factors that could be associated with a poor performance of materials in OFETs would be (i) a particularly low intrinsic charge carrier mobility of the material, (ii) the poor injection of charge carriers at the electrodes and (iii) trapping of charge carriers in the accumulation region of the transistor channel (*i.e.*, at the interface between the gate dielectric and the active layer).

Regarding the intrinsic carrier mobility, we calculated the values of transfer integrals for the specific OEG-BTBT structure confirmed by Raman scattering experiments and simulations. The transfer integrals in OEG-BTBT are 2–3 times smaller than in alkyl-substituted BTBT derivatives, which would reduce the expected carrier mobility in OEG-BTBT by an order of magnitude. The observed values for OEG-BTBT are, however, four to five orders of magnitude smaller than the reported 10 cm^2^ V^−1^ s^−1^ in OFET devices based on thermally evaporated alkyl-substituted BTBT films.^9^

We can also exclude an unfavourable texture of the films, as the XRD data reveal that the high-mobility plane of OEG-BTBT is aligned along the channel of the transistor. This finding is supported by atomic force microscopy measurements, that showed flat molecular layers comprising essentially upright-standing molecules (as inferred from the layer thickness). These layers are oriented parallel to the surface of the device channel, which is preferential for charge transport. The AFM surface topography measurements also show that under optimized deposition conditions (*T*_substrate_ = 60 °C) large lateral grain sizes (>1 μm) are observed, which is on the same length scale as the device channel length (Fig. 3(b) and (d)), indicating a low density of grain boundaries along the charge transport direction. Moreover, the insight from the AFM and XRD studies in combination with previous results suggest Stranski–Krastanov type film growth with continuous OEG-BTBT layers underneath the crystallites observed in AFM.^42^ These considerations suggest that a poor intrinsic carrier mobility within the studied thin films cannot be held responsible for the subpar OFET performance.

To address the role played by potentially mediocre charge carrier injection/extraction at the source and drain contacts, we varied the electrode metal and tested a metal electrode surface modification by self-assembled monolayer. Especially the latter leads to an electrode work-function ideally matching the ionization energy of OEG-BTBT (*ϕ*_Au+PFBT_ = 5.6 eV *vs.* IE_OEG-BTBT_ = 5.54 ± 0.03).^16,48^ Nevertheless, no relevant improvement of the device performance was obtained for the Au + PFBT electrode, suggesting that contact-related effects are also not the culprit.

This leaves carrier trapping as the most likely cause of the poor performance of OEG-BTBT OFETs. This notion is supported by the bias stress measurements, which indicated the presence of significant charge trapping at the OSC/dielectric interface in OEG-BTBT devices (Fig. 5(c) and (d)). As discussed previously, the high ionization energy of OEG-BTBT (IE = 5.54 ± 0.03 eV) is similar to that of other alkyl- and alkoxy-substituted BTBT derivatives, which makes them susceptible to water-induced hole trapping.^49^ Notably, in alkyl-substituted BTBT derivatives this has been successfully mitigated by passivating the surface of the dielectric using hydrophobic SAMs.^9,50^ This is attributed to a severely reduced water concentration at the interface, when combining the hydrophobic SAMs with an organic semiconductor bearing hydrophobic side chains. However, as described in previous sections, the modification of OEG-BTBT transistor devices with ODTS monolayers fell short of eliminating degradation effects induced by the gate bias, even though the passivation resulted in a noticeable improvement in device performance. We propose that this is caused by the inherent hydrophilicity of the oligo(ethylene glycol) side chains which leads to a significantly increased concentration of water molecules in the bulk of the OSC. Consequently, this leads to an increased number of potential trap sites at the interface with the dielectric, which cannot be fully eliminated only by passivating the SiO_2_ surface, since water molecules can migrate from the bulk to the interface. To resolve this problem, alternative approaches might be required, such as the use of molecular additives, to reduce the amount of residual water in the bulk of the OSC.^51^

Indeed, previous studies have reported that OEG-BTBT exhibits a notable affinity towards humidity in ambient air.^16^ As reported in this study, the high sensitivity to water is observed even in low-humidity environments (H_2_O < 5 ppm). This affinity for water together with the high ionization energy promotes charge trapping within the charge accumulation region at the dielectric interface, which are likely to be the key factors causing the observed performance limitations in OEG-BTBT-based OFET devices.

## Conclusion

In this study, we report an investigation of the charge transport properties of a novel BTBT derivative with hydrophilic oligo(ethylene glycol) side chains. Transfer integrals were calculated and compared to other BTBT derivatives. We employed a combined low-frequency Raman spectroscopy and DFT calculations approach to identify the polymorphic crystal form of OEG-BTBT. The thin film morphology of OEG-BTBT films was evaluated using AFM and XRR analysis as a function of the substrate temperature during the physical vapor deposition, revealing the thin film growth parameters yielding optimal film morphology for OFET applications.

Despite the intrinsic electronic properties of OEG-BTBT being similar to other high-mobility derivatives of BTBT, we observed a subpar performance of OEG-BTBT-based OFET devices, manifested by a low effective charge carrier mobility, a high threshold voltage, and a large hysteresis. Rather than simply considering this as a failed experiment, we used an extensive toolbox of techniques to identify the origin of this low performance since it is important to better understand the molecular design rules for charge transport in small molecule semiconductors with such polar side chains. We ruled out a low intrinsic mobility and problems with carrier injection as its primary cause. Rather, we identified bias stress effects that lead to the degradation of the OSC/dielectric interface when operating the OEG-BTBT transistor devices at higher gate voltages.

We propose that these effects are linked to the hydrophilic nature of oligo(ethylene–glycol) side chains, which leads to an increased concentration of residual water molecules in the bulk of OEG-BTBT film. Consequently, this affinity for water alongside the high ionization energy of OEG-BTBT promotes hole trapping in the charge accumulation region at the dielectric interface. These characteristics impose substantial limitations on the performance of OEG-BTBT-based OFET devices and underscore the challenges associated with employing OEG-BTBT in electronic device applications.

## Data availability statement

The data supporting this article will be made available upon acceptance of the manuscript on Apollo, the University of Cambridge institutional data repository.

## Conflicts of interest

There are no conflicts to declare.

## Supplementary Material

MA-005-D4MA00594E-s001
