# Supplementary material for: Impact of hydrophilic side chains on the thin film transistor performance of a benzothieno–benzothiophene derivative
Source: Mater Adv. 2024 Jul 10;5(15):6285–94. doi: 10.1039/d4ma00594e (PMC11284759; doi:10.1039/d4ma00594e)
Supplement: MA-005-D4MA00594E-s001 [file MA-005-D4MA00594E-s001.pdf]

Electronic Supplementary Material (ESI) for Materials Advances.
This journal is © The Royal Society of Chemistry 2024

Supporting Information

Impact of Hydrophilic Side Chains on the Thin Film Transistor Performance of a Benzothieno-Benzothiophene Derivative

Mindaugas Gicevičius[a*], Ann Maria James[b], Lukas Reicht[b], Nemo McIntosh[c], Alessandro Greco[d], Lamia Fijahi[e], Félix Devaux[f], Marta Mas-Torrent[e], Jérôme Cornil[c], Yves Henri Geerts[f,g], Egbert Zojer[b], Roland Resel[b], Henning Sirringhaus[a*]

[a] Optoelectronics Group, Cavendish Laboratory, University of Cambridge, JJ Thomson Avenue, Cambridge CB3 0HE, United Kingdom
[b] Institute of Solid State Physics, NAWI Graz, Graz University of Technology, Petersgasse 16, 8010 Graz, Austria
[c] Laboratory for Chemistry of Novel Materials, University of Mons, 7000 Mons, Belgium
[d] Max Planck Institute for Polymer Research, 55128 Mainz, Germany
[e] Institut de Ciència de Materials de Barcelona, ICMAB-CSIC, Campus de la UAB, 08193 Bellaterra, Spain
[f] Laboratoire de Chimie des Polymères, Faculté des Sciences, Université Libre de Bruxelles (ULB), CP 206/1, Boulevard du Triomphe, 1050 Bruxelles, Belgium
[g] International Solvay Institutes of Physics and Chemistry, Université Libre de Bruxelles, 1050 Bruxelles, Belgium

* Corresponding author, e-mail: mg980@cam.ac.uk, hs220@cam.ac.uk

Table of Contents

UV/Vis absorption spectrum of OEG-BTBT 2
OEG-BTBT thin film transistor device architecture 3
ODTS SAM effect on OEG-BTBT transistor transfer characteristics 4
X-Ray reflectivity measurements ODTS self-assembled monolayer 5
Energy cutoff and k-mesh convergence 6
Raman spectrum for the fully optimized cell 8
Anharmonicities 9
References 11

UV/Vis absorption spectrum of OEG-BTBT

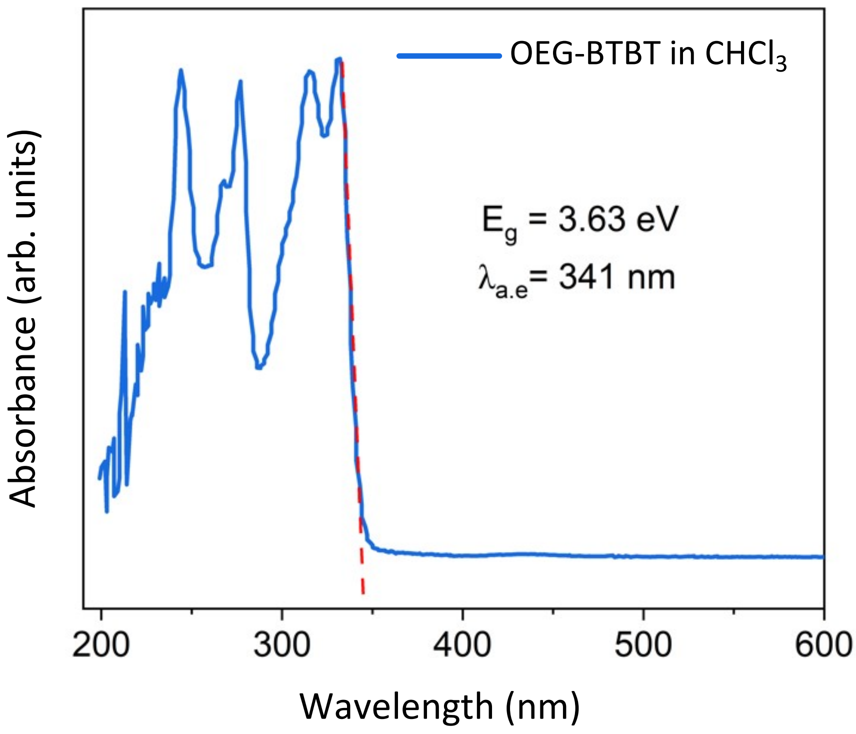

Figure S1: (a) UV-Vis absorption spectrum of OEG-BTBT solution in chloroform.

OEG-BTBT thin film transistor device architecture

(a) (b)

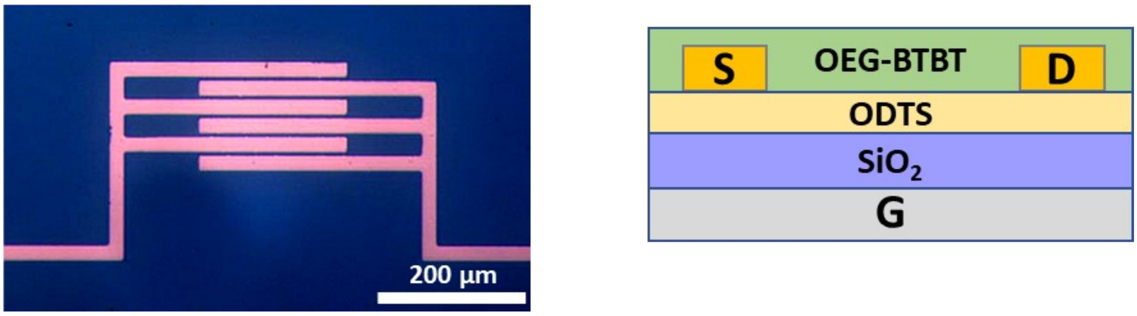

Figure S2: (a) Interdigitated source and drain electrode layout: channel length is 5 µm, width - 1000 µm. (b) Schematics of bottom-gate/bottom-contact device architecture for OEG-BTBT-based OFET devices.

ODTS SAM effect on OEG-BTBT transistor transfer characteristics

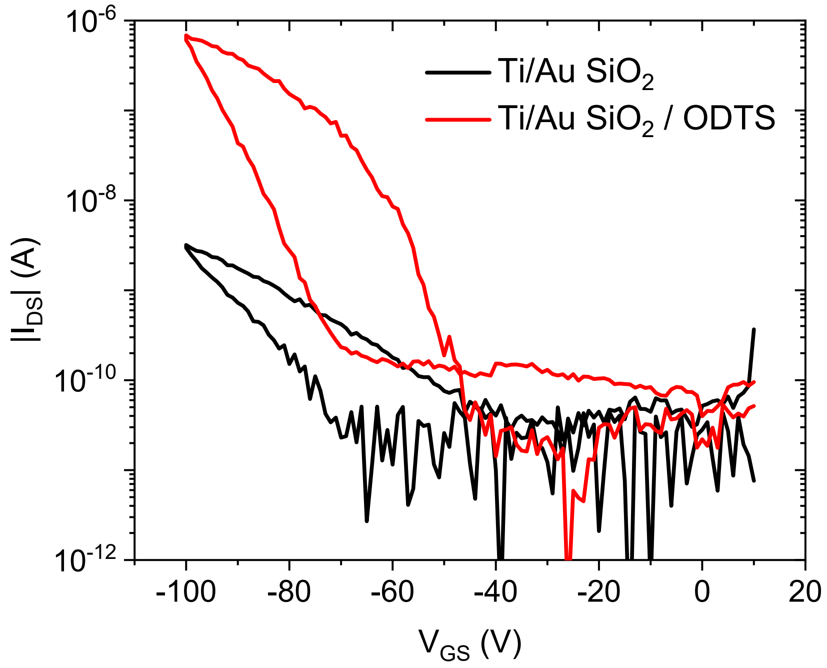

Figure S3: The effect of SiO_2 dielectric modification with n-octadecyltrichlorosilane (ODTS) self-assembled monolayer on transfer characteristics in saturation regime of OEG-BTBT-based OFET devices bearing Ti/Au contacts (L = 5 µm).

X-Ray reflectivity measurements ODTS self-assembled monolayer

X-ray reflectivity data of the used substrates: 300 nm thermally oxidized silicon (grey curve) and ODTS-treated thermally oxidized silicon (black curve). Fitting of the experimental data (red curves) reveal a mass density of 2.3 g cm^{-3} and a surface roughness of 0.37 nm for the untreated substrate. For the ODTS-treated substrate a mass density was found to be between 1.3 g cm^{-3} and 1.4 g cm^{-3}, and a film thickness of 1.6 nm with a surface roughness of 0.3 nm was determined.

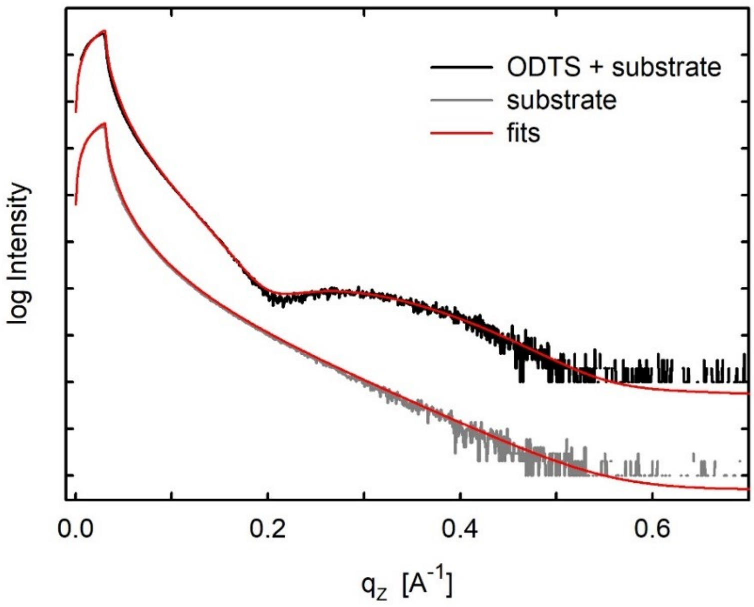

Figure S4: X-ray reflectivity measurements and corresponding fits of n-octadecyltrichlorosilane (ODTS) self-assembled monolayer deposited on SiO_2 substrate.

Energy cutoff and k-mesh convergence

For the calculation of the Raman spectrum with DFT, the energy cutoff and k-mesh were chosen such that the total energy per atom is converged to below 0.5 meV. To that end, we performed calculations of the total energy per atom with energy cutoffs of 400 eV, 500 eV, 600 eV, 700 eV, 800 eV, 900 eV, 1000 eV, 1100 eV and 1200 eV at a 1×2×2 k-mesh. The results are shown in Figure S5. Additionally, to determine the necessary k-point sampling of the first Brillouin zone for obtaining the charge densities, calculations with 1×2×2, 2×5×5, 3×7×7 and 4×10×9 k-meshes were performed at a cutoff energy of 400 eV. The resulting energies are presented in Figure S6. These data show that the energy per atom is converged to below 0.5 meV for an energy cutoff of 900 eV and a k-mesh of 1×2×2.

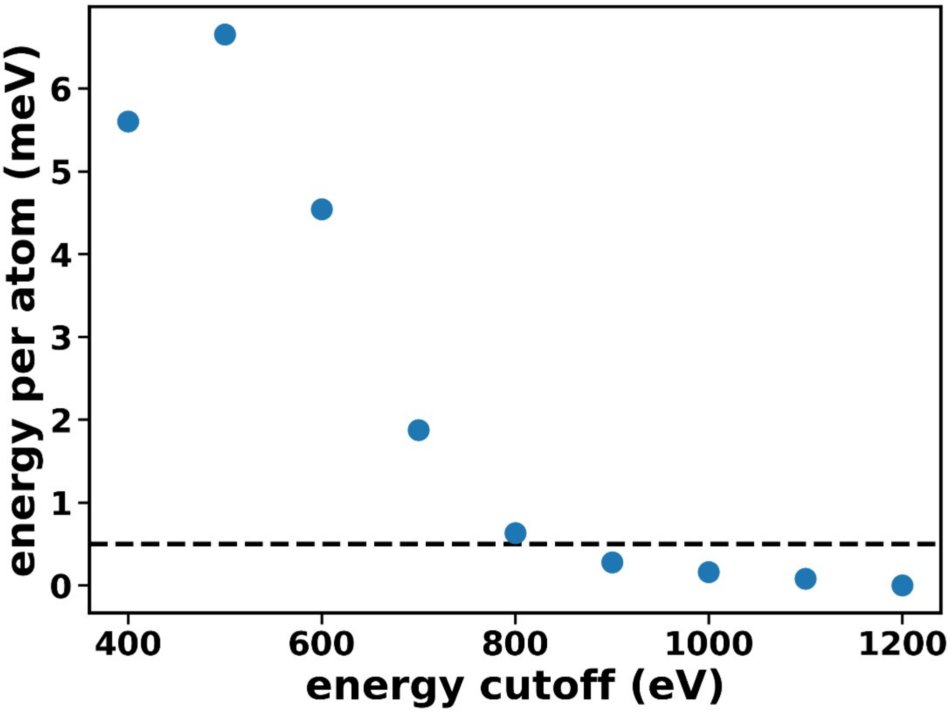

Figure S5: Convergence test of the energy per atom in terms of energy cutoff. The shown total energies per atom at the equilibrium geometry are relative to the energy at an energy cutoff of 1200 eV. The convergence criterion of 0.5 meV per atom is shown as a black dashed line.

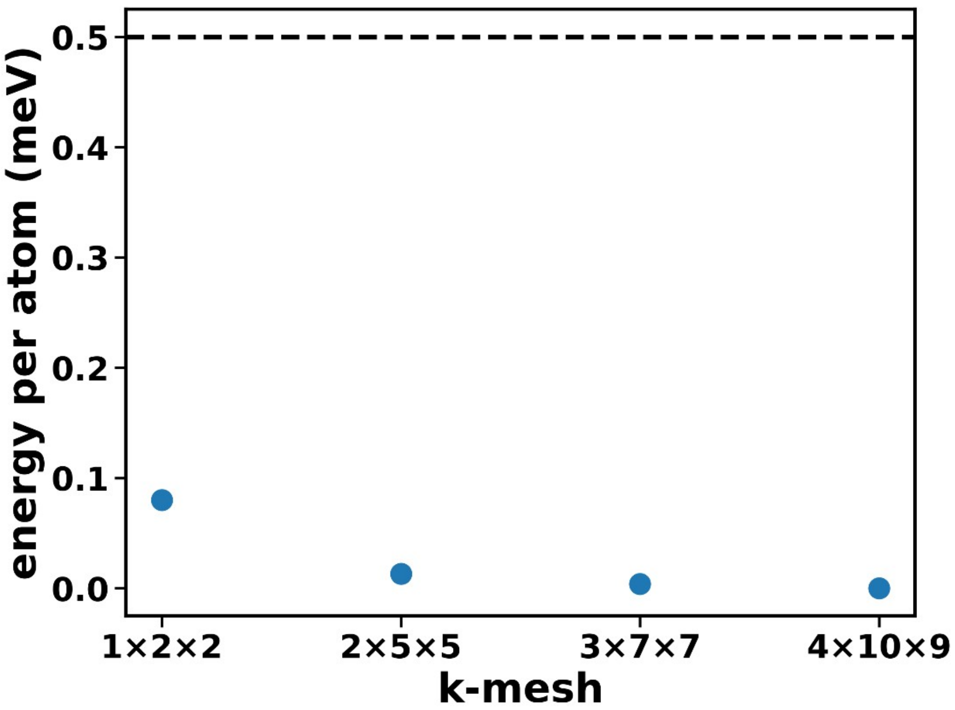

Figure S6: Convergence test of the energy per atom in terms of k-mesh. The shown total energies per atom at the equilibrium geometry are relative to the energy with a k-mesh of 4×10×9. The convergence criterion of 0.5 meV per atom is shown as a black dashed line.

Raman spectrum for the fully optimized cell

In addition to calculating the Raman spectrum for the experimental unit cell of OEG-BTBT, we also fully relaxed the lattice parameters. This yielded the following lengths and angles between the unit cell vectors: 18.423 Å, 7.587 Å, 8.253 Å, 90°, 100.419°, and 90°. Calculating the Raman spectrum for that relaxed unit cell resulted in a clearly worse agreement with the experimental spectrum, as shown in Figure S7. We attribute this to thermal expansion effects neglected in the DFT-relaxed cell, which corresponds to a T→0K structure. The resulting too small unit cell causes a shift of all Raman peaks to too high wavenumbers.

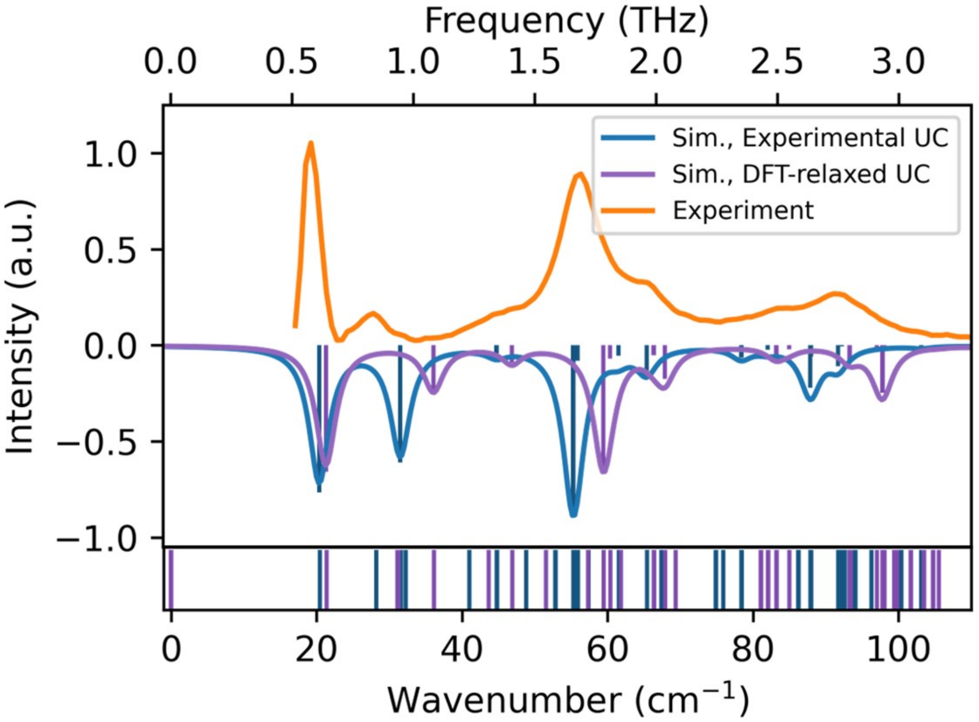

Figure S7: Experimental (orange) and simulated Raman spectra of OEG-BTBT. The calculations were performed for the experimental (blue) and fully DFT relaxed (purple) unit cells. For the simulated spectra, in the upper panel (downwards-pointing spectra), Raman-active modes are indicated by bars scaled with their Raman intensities. The lower panel illustrates the frequencies of all modes (including Raman-active and Raman-inactive ones).

Anharmonicities

Schweicher et al. found strong anharmonicities in C8-BTBT-C8 and C8-DNTT-C8, which prompted us to investigate this matter also for OEG-BTBT.[1] We analyzed possible anharmonicities of the low-frequency modes in the same fashion as we did in the SI of reference 2:[2] We displaced the structure along the mass-weighted eigenvectors of an eigenmode. The magnitude of this displacement is given by the displacement distances Q. It was chosen to range from -3 to +3 Å with 60 steps. A single point calculation was performed for each displacement distance Q with the data shown as blue dots in Figure S8. They are compared to the shapes of the harmonic approximation of the PES derived from the harmonic frequencies of the eigenmodes ω_λ, whereby the composite index λ denotes the band index and the reciprocal lattice vector. These frequencies were obtained from the lattice dynamics calculations using phonopy as described in the main text.[3] The energies as a function of the displacement distance Q are then given by

$$E_\lambda(Q) = \frac{\omega_\lambda^2 \mu_\lambda}{2} Q^2 \qquad \text{(S1)},$$

where μ_λ is the effective mass of the mode λ. This equation corresponds to a term in the Hamiltonian of the quantum harmonic oscillator equation, when writing it as a superposition of independent one-dimensional harmonic oscillators. The effective mass μ_λ was calculated as described in the SI of reference [4]

$$\mu_\lambda = \frac{\sum_i^N |e_\lambda^i|^2}{\sum_i^N m_i^{-1} |e_\lambda^i|^2} \qquad \text{(S2)},$$

with the eigenvector e_i and the mass of each atom m_i. The sum runs over all atoms i. $E_\lambda(Q)$ is shown as a solid orange line in Figure S8. Its minimum is aligned with the energy at the equilibrium structure and defined as the zero of the energy axis. In the following, we focus on bands 4 to 7 at the Γ-point, which are the lowest optical bands and at the same time at Γ the lowest bands with non-zero energy. As can be seen in Figure S8 for modes 4 and 5, the explicitly calculated $E_\lambda(Q)$ curves lie close to the harmonic energies. For mode 6, the displaced structure at Q=-0.1 has a lower energy than the Q=0 structure by 0.035 meV. It appears that the explicitly calculated energy curve is shifted with respect to the curve for the harmonic calculation, which is similar to the observations in the SI of reference 2 for an α-quinacridone crystal.[2] We attribute this small shift to the relaxation procedure, which for the chosen convergence criterion apparently yields an optimized structure that does not exactly correspond to the minimum with respect to Mode 6. We note in passing, that in both cases (OEG-BTBT and quinacridone), the mode corresponds to a rotation of the molecule. Most importantly, this shift in the origin of the displacement axis has only a negligible impact on the curvature of the potential and, thus, the eigenfrequency. As reference energy, we consider 25 meV, which is k_BT at room temperature and in a classical picture equals the average total energy of a phonon mode. For all modes discussed so far, there are hardly any deviations between the explicitly calculated energy of the displaced structure and the energy obtained from the harmonic oscillator model at this reference energy (safe the rigid shift of the potentials for mode 6). The situation is different for mode 7 (a bending motion of the side chains): At very small displacements the orange line and the blue data points coincide, but already at displacements associated with an energy of 25 meV there are significant differences in the displacements suggesting non-negligible anharmonicities, e.g., at room temperatures. Still, this has no impact on the Raman spectra, as of the considered modes only modes 4 (at 20.45 cm^{-1}) and 6 (at 31.58 cm^{-1}) are Raman active, whereas mode 5 (at 28.2 cm^{-1}) and mode 7 (at 32.24 cm^{-1}) are not.

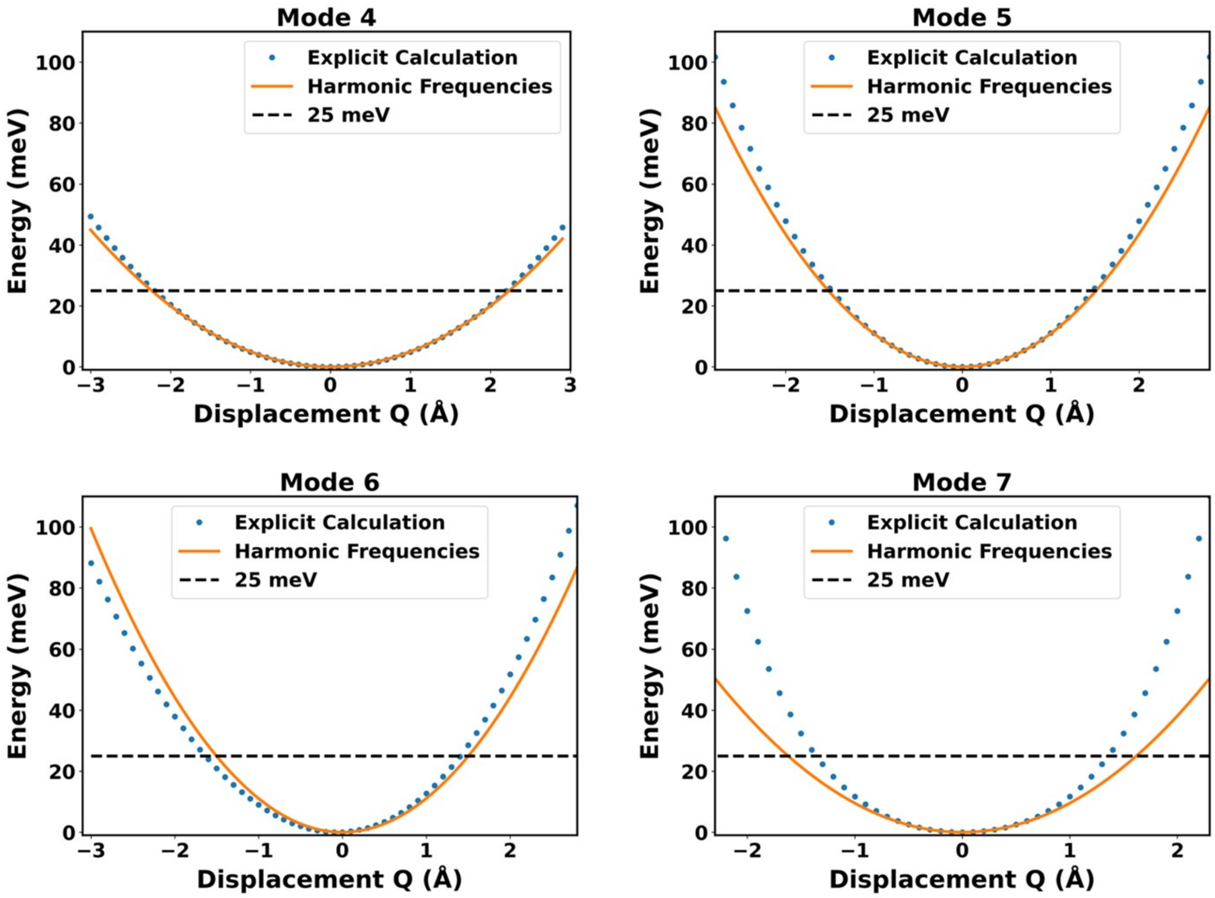

Figure S8: Energy (relative to the energy of the equilibrium structure) versus eigenmode displacement Q for modes 4 to 7. The blue dots show the explicitly calculated energies, while the orange lines show the parabolic potential energy surfaces derived from eigenfrequencies within the harmonic approximation. The dashed black line indicates the thermal energy at room temperature (25 meV). We present here calculations for the experimental unit cell and note in passing that equivalent results have been obtained for the DFT-relaxed unit cell.

References

[1] G. Schweicher, G. D'Avino, M. T. Ruggiero, D. J. Harkin, K. Broch, D. Venkateshvaran, G. Liu, A. Richard, C. Ruzié, J. Armstrong, A. R. Kennedy, K. Shankland, K. Takimiya, Y. H. Geerts, J. A. Zeitler, S. Fratini, H. Sirringhaus, *Adv. Mater.* **2019**, *31*, 1902407.
[2] L. Legenstein, L. Reicht, T. Kamencek, E. Zojer, *ACS Mater. Au* **2023**, *3*, 371.
[3] A. Togo, *J. Phys. Soc. Japan* **2023**, *92*, DOI 10.7566/JPSJ.92.012001.
[4] J. George, R. Wang, U. Englert, R. Dronskowski, *J. Chem. Phys.* **2017**, *147*, DOI 10.1063/1.4985886.
